# Regulation of growth hormone secretion by (pro)renin receptor

**DOI:** 10.1038/srep10878

**Published:** 2015-06-03

**Authors:** Yuji Tani, Shozo Yamada, Naoko Inoshita, Yukio Hirata, Masayoshi Shichiri

**Affiliations:** 1 Department of Endocrinology, Diabetes and Metabolism, Kitasato University School of Medicine, Kanagawa, Japan; 2 Department of Hypothalamic and Pituitary Surgery, Toranomon Hospital and Okinaka Memorial Institute for Medical Research, Tokyo, Japan; 3 Department of Pathology, Toranomon Hospital and Okinaka Memorial Institute for Medical Research, Tokyo, Japan; 4 Institute of Biomedical Research and Innovation Hospital, Hyogo, Japan

## Abstract

(Pro)renin receptor (PRR) has a single transmembrane domain that co-purifies with the vacuolar H^+^-ATPase (V-ATPase). In addition to its role in cellular acidification, V-ATPase has been implicated in membrane fusion and exocytosis via its Vo domain. Results from the present study show that PRR is expressed in pituitary adenoma cells and regulates growth hormone (GH) release via V-ATPase-induced cellular acidification. Positive PRR immunoreactivity was detected more often in surgically resected, growth hormone-producing adenomas (GHomas) than in nonfunctional pituitary adenomas. GHomas strongly expressing PRR showed excess GH secretion, as evidenced by distinctly high plasma GH and insulin-like growth factor-1 levels, as well as an elevated nadir GH in response to the oral glucose tolerance test. Suppression of PRR expression in rat GHoma-derived GH3 cells using PRR siRNA resulted in reduced GH secretion and significantly enhanced intracellular GH accumulation. GH3 treatment with bafilomycin A1, a V-ATPase inhibitor, also blocked GH release, indicating mediation via impaired cellular acidification of V-ATPase. PRR knockdown decreased Atp6l, a subunit of the Vo domain that destabilizes V-ATPase assembly, increased intracellular GH, and decreased GH release. To our knowledge, this is the first report demonstrating a pivotal role for PRR in a pituitary hormone release mechanism.

(Pro)renin receptor (PRR) was first identified as a 350-amino acid protein with a single transmembrane domain^1^. Prorenin binds to this putative receptor with a higher affinity than renin to activate ERK1/2 independently from angiotensin II (AngII) generation^2^,^3^,^4^, and is also capable of initiating AngII-dependent effects, although less potently than renin^1^,^5^. In contrast to initial expectations, however, PRR rarely acts as a cell surface receptor for extracellular renin/prorenin molecules, because they easily undergo proteolytic cleavage to excise out extracellular domains^6^,^7^,^8^. The transmembrane domain of PRR was found to be identical to an intracellular protein associated with the vacuolar H^+^-ATPase (V-ATPase)^9^, named vacuolar H^+^-ATPase-associated protein 2 (ATP6ap2). V-ATPase, a large multi-subunit complex comprising V1 and Vo, is a major proton pump that controls proton homeostasis in eukaryotic cells, and regulates the pH of intracellular compartments^10^. A V1 catalytic domain that hydrolyzes ATP is composed of eight subunits (A–H), while a Vo domain involved in proton translocation contains six subunits a, d, e, c, c′, and c″. Genetic ablation of Atp6ap2 down-regulates Vo c subunit (Atp6l) and selectively affects stability and assembly of the Vo domain, thereby compromising vesicular acidification^11^,^12^. The resulting acidic environment is crucial for many biological processes, such as intracellular trafficking and coupled transport of small molecules^13^,^14^. PRR also interacts with other signaling proteins independently from AngII generation, such as Wnt receptors^15^,^16^,^17^ and the transcription factor promyelocytic leukemia zinc finger (PLZF)^18^,^19^,^20^. PLZF, originally identified as the fusion partner of the retinoic acid receptor α^21^, undergoes nuclear translocation following renin stimulation and represses transcription of PRR itself, as well as activates PI3K-p85α^18^.

PRR is ubiquitously expressed in a variety of tissues^1^,^22^,^23^ and involved in cardiovascular and renal diseases in experimental models^20^,^24^. PRR mRNA colocalizes with GH and ACTH^25^, while its protein is abundantly present in the human anterior lobe^26^. All RAS components coexist within the secretory granules of all cell types of the rat anterior pituitary^27^, as well as lactotropes in normal human pituitary and PRL-secreting adenomas^28^,^29^. In the human hypothalamus and pituitary, PRR protein is localized to the paraventricular and supraoptic nuclei, as well as in anterior pituitary cells^26^. Despite our knowledge of systemic and central distribution of PRR and RAS components to date, very limited information is available for their central roles in humans. Further studies are needed to determine whether PRR/Atp6ap2 regulates hormone secretion.

In GHomas, gain-of-function point mutations of the Gs protein, termed gsp, lead to constitutive adenylyl cyclase induction and are thought to promote GH secretion via GH-releasing hormone^30^,^31^. Gain-of-function point mutations also account for 30–40% of GHomas^32^,^33^,^34^. However, the pathogenic mechanisms underlying excessive GH production in the remaining GHomas are unknown. In addition, hormonal release mechanisms in pituitary tumors remain poorly understood.

## Results

### PRR expression in human pituitary adenomas

**Figure 1:**
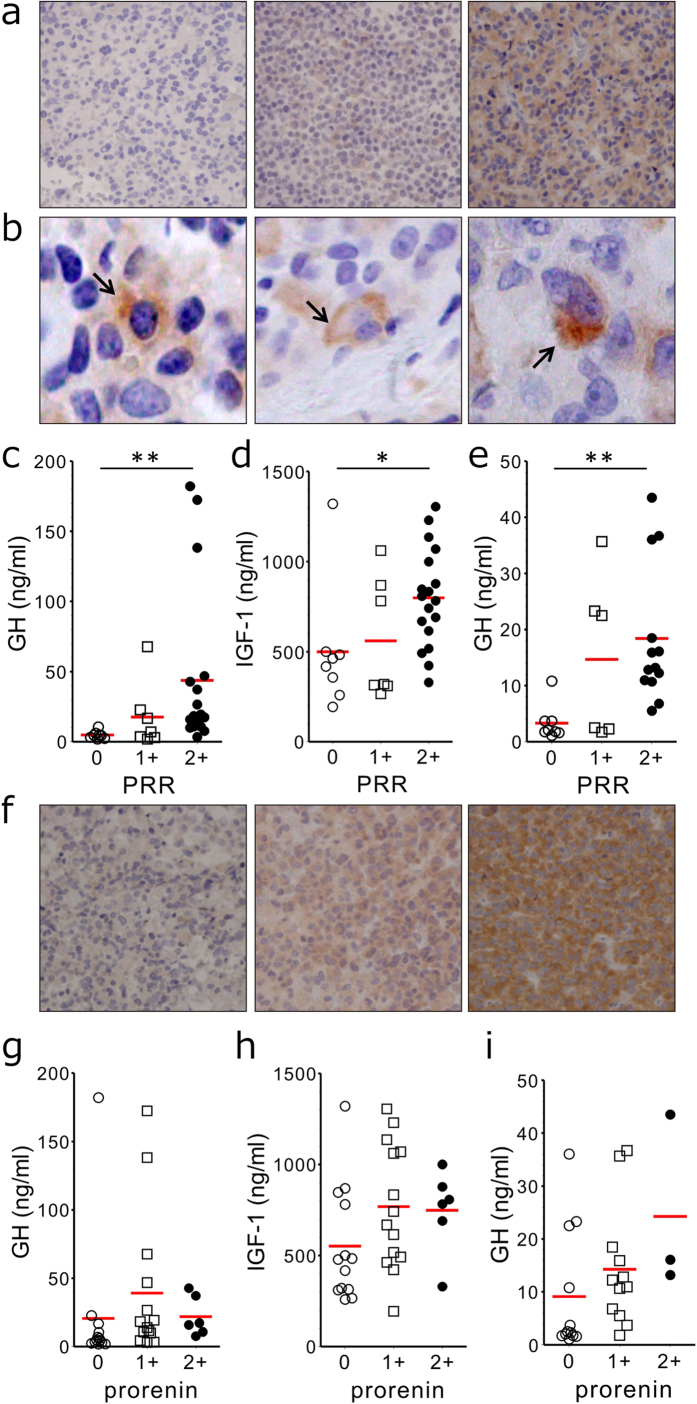
Immunoreactive PRR and prorenin in GHomas and GH hormonal activities. **a**, Immunohistochemistry of human GHomas with an anti-PRR antibody. Paraffin sections of surgically resected GHomas were stained with an anti-PRR antibody, and PRR immunoreactivity was semi-quantitatively graded (magnification × 100): left panel, negative (0); middle panel, weakly positive (1+); right panel, strongly positive (2+). **b**, Localization of immunoreactive PRR within GHomas: Golgi-like (left panel), lysosome-like (middle panel), and granular pattern (right panel) localizations are indicated with arrows (magnification × 400). **c–e,** Association of peripheral GH hormonal activity with PRR immunoreactivity in resected GHomas. Baseline serum GH levels (**c**) baseline IGF-1 levels (**d**) and nadir GH levels in response to an OGTT (**e**) in patients with GHomas showing respective immunoreactive PRR intensities (0, 1+, or 2+) are shown. The Dunn’s post-hoc test compared with each group. *P < 0.05 and **P < 0.01. **f**, Anti-prorenin immunohistochemistry of human GHomas. Paraffin sections of surgically resected GHoma were stained with anti-prorenin antibody, and prorenin-like immunoreactivities were semi-quantitatively graded (magnification × 100): left panel, negative to weakly positive (0); middle panel, moderately positive (1+); right panel, strongly positive (2+). **g–i**, No significant association of peripheral GH hormonal activity with prorenin-like immunoreactivity in resected GHomas. Baseline serum GH levels (**g**) baseline IGF-1 levels (**h**) and nadir GH levels in response to an OGTT (i) in patients with GHomas showing respective immunoreactive prorenin intensities (0, 1+, and 2+). The Dunn’s post hoc test compared with each group.

We first analyzed whether PRR protein was expressed in human functioning and non-functioning pituitary adenomas using immunohistochemical analysis. Positive immunostainings for PRR were observed in 9 of 29 (31%) nonfunctional pituitary adenomas, 25 of 33 (76%) GHomas, 7 of 14 (50%) ACTH-secreting pituitary adenomas, and 1 of 7 (14%) TSH-secreting pituitary adenomas. Of a total of 33 patients with acromegaly (13 were male, 20 were female), 15 were treated with primary medical therapy prior to surgery (80% somatostatin analogs, 26% dopamine agonists, 7% combined therapy). Assessment of tumor size at surgery showed 30 macroadenomas and 3 microadenomas. Because the majority of GHomas showed positive immunostaining for PRR, we semi-quantitatively evaluated intensities of their immunoreactivities. Eight cases (24%) were negative, seven cases (21%) were weakly positive, and 18 cases (55%) were strongly positive for PRR (Fig. 1a). Immunoreactive PRR in GHomas appeared to distribute either in the Golgi apparatus or around lysosomes, or alternatively as granular particles (Fig. 1b).

### Association of PRR expression in GHomas with GH hormonal activity

To determine whether hormonal activities in patients with acromegaly are related to PRR expression in tumor tissues, the association of PRR expression with circulating GH, IGF-1 and nadir GH in response to OGTT was studied in all 33 acromegalic patients. Serum GH (43.7 ± 13.5 ng/mL) and IGF-1 (798.4 ± 64.7 ng/mL) levels of 18 cases who showed strongly positive PRR expression in GHomas were both significantly (*P* < 0.001; *P* < 0.05) greater than 8 cases with negative PRR expression (GH, 4.8 ± 1.0 ng/mL; IGF-1, 499.9 ± 123.4 ng/mL) (Fig. 1c,d). Serum GH (17.6 ± 8.9 ng/mL) and IGF-1 (560.9 ± 125.5 ng/mL) levels in seven cases with weakly positive PRR expression were greater than those with negative expression, but the differences did not reach statistical significance (Fig. 1c,d). The nadir GH levels in response to OGTT (18.4 ± 3.4 ng/mL) of 18 patients with strongly positive PRR expression were also significantly (*P* < 0.01) greater than those with negative PRR expression (3.3 ± 1.1 ng/mL) (Fig. 1e).

### No significant association of prorenin expression with circulating GH and IGF-I in GHomas

We next determined whether the expression of an endogenous ligand for PRR on plasma membrane was associated with GH activity. GHomas were classified into three categories with respect to intensity of prorenin immunoreactivity. Thirteen cases (39%) were either negative- or weakly positive, 14 cases (42%) were moderately positive, and six cases (18%) were strongly positive for prorenin (Fig. 1f). There were no significant differences in GH, IGF-1 or nadir GH levels in response to OGTT between any of the two groups (Fig. 1g,h,i).

### Expression of PRR and intracellular acidification in GH3 cells

**Figure 2:**
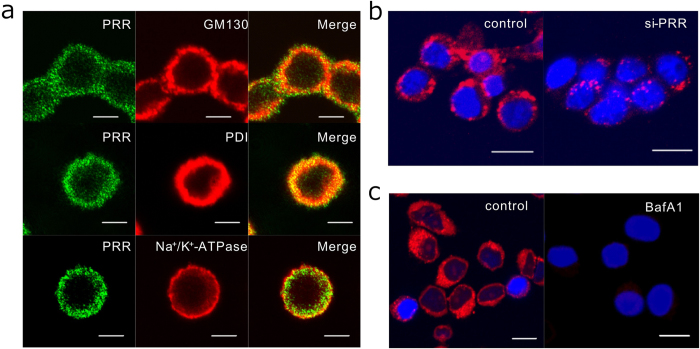
Subcellular PRR localization and acidic organelle in GH3 cells by immunofluorescence analysis. **a** Immunofluorescent st**a**inings using antibodies against PRR (*green*) (left panels), GM130, PDI, and Na^+^/K^+^-ATPase (*red*) (middle panels), and the merged images (right panels) are shown. Scale bar, 5 μm. **b** Acidic organelles were labeled using LysoTracker (*red*) in GH3 cells transfected with scramble control siRNA (control) or with PRR si-RNA (si-PRR) for 30 min. Scale bar, 10 μm. **c** Acidic organelles were detected in GH3 cells pretreated without (control) or with bafilomycin A1 (BafA1, 100 nM) for 30 min. Scale bar, 10 μm.

The above results, which revealed a close association between abundant PRR expression and excessive GH secretion in GHomas, prompted us to examine PRR expression in a rat GHoma cell line, GH3. Immunofluorescence demonstrated abundant PRR expression localized intracellularly in GH3 cells. The positive fluorescence merged with the Golgi marker GM130, and the endoplasmic reticulum marker PDI, but only partially with the membrane marker Na^+^/K^+^-ATPase α1 (Fig. 2a), suggesting predominantly subcellular localization of PRR in intracellular compartments, rather than in the plasma membrane.

Because the C-terminal part of PRR constitutes an adaptor protein of V-ATPase^6^, we examined whether PRR expression was related to intracellular acidification in GH3 cells. Transfection of GH3 cells with siRNA against rat PRR significantly decreased immunofluorescence of intracellular acidic organelles (Fig. 2b). Treatment of GH3 cells with an inhibitor of V-ATPase, bafilomycin A1, completely abolished the immunofluorescent signals of acidic organelles (Fig. 2c).

### Relationship between PRR expression and GH secretion in GH3 cells

**Figure 3:**
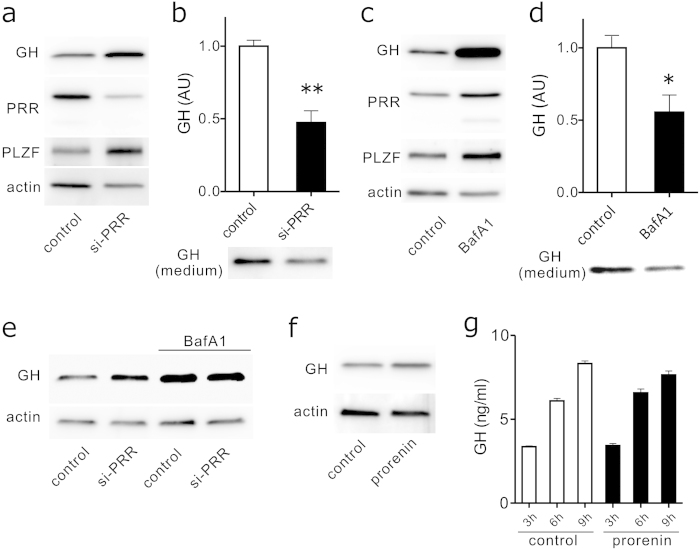
Effect of PRR siRNA transfection and bafilomycin A1 treatment on intracellular GH accumulation and GH release in GH3 cells. **a** GH3 cells were transfected with either scramble control siRNA (control) or PRR si-RNA (si-PRR) for 48 h, and cell lysates were subjected to immunoblotting using anti-GH, anti-PRR, anti-PLZF, and anti-actin antibodies. **b** Conditioned medium from cultured GH3 cells transfected with either scramble control siRNA (control) or PRR si-RNA (si-PRR) for 48 h was extracted and immunoblotted with an anti-GH antibody. A representative immunoblot (lower panel) and quantification of GH protein levels normalized to WST-1 absorbance (n = 3, upper panel) are shown. **P < 0.01. **c** GH3 cells were treated without (control) or with bafilomycin A1 (BafA1, 100 nM) for 24 h, and cell lysates were subjected to immunoblotting using anti-GH, anti-PRR, anti-PLZF, and anti-actin antibodies. **d** Conditioned medium from cultured GH3 cells treated without (control) or with bafilomycin A1 (BafA1, 100 nM) for 24 h, and cell lysates were subjected to immunoblotting using an anti-GH antibody. A representative immunoblot (lower panel) and quantification of GH protein levels normalized to WST-1 absorbance (n = 3, upper panel) are shown. *P < 0.05. **e** GH3 cells were transfected with either scramble control siRNA (control) or PRR si-RNA (si-PRR) for 48 h, then treated without or with bafilomycin A1 (BafA1, 100 nM) for 9 h, and cell lysates were subjected to immunoblotting using anti-GH and anti-actin antibodies. **f** GH3 cells were treated without (control) or with recombinant human prorenin (5 nM) for 24 h, and cell lysates were subjected to immunoblotting using anti-GH and anti-actin antibodies. **g** Subconfluent GH3 cells were replaced with serum-free medium without (control) or with prorenin (5 nM), incubated for the indicated times and GH levels in each conditioned media were measured by GH ELISA (n = 4). Each column and bar represents mean ± SEM. Full-length blots were shown in supplementary figures 1 and 2.

To determine whether PRR expression influences GH secretion from GH3 cells, we performed immunoblot analysis using whole GH3 cell lysates and GH ELISA of cultured conditioned media of GH3 cells transfected with or without rat PRR siRNA. PRR suppression increased both GH and PLZF protein levels within the cells (Fig. 3a), while significantly (*P* < 0.01) reducing immunoreactive GH released into the culture supernatant (Fig. 3b). To determine whether disruption of intracellular acidification via V-ATPase affects GH secretion, we studied the effect of bafilomycin A1 on GH secretion. Bafilomycin A1 treatment increased both GH and PLZF immunoreactivities within cell lysates (Fig. 3c), and significantly (*P* < 0.05) decreased GH secretion into the culture media (Fig. 3d). We next determined whether simultaneous treatment with Bafilomycin A1 and PRR siRNA exerts additive effects. GH3 cells preteated with si-PRR or scramble control were incubated with or without bafilomycin A1 and GH expression in the cell lysates was detected by Western blot. Bafilomycin A1 did not show any appreciable effect on intracellular GH accumulation (Fig. 3e).

To negate the possible involvement of classical RAS pathway in regulating GH secretion, we studied the effect of recombinant human prorenin on GH3 cells. Addition of prorenin did not affect intracellular GH protein levels (Fig. 3f) nor GH protein released into culture supernatant (Fig. 3g). Prorenin did not modulate PLZF or p42/44 ERK1/2 expression levels (data not shown). These results argue against the role for a classical RAS pathway in the regulation of GH release.

### PRR-PLZF transduction pathway does not affect GH expression

**Figure 4:**
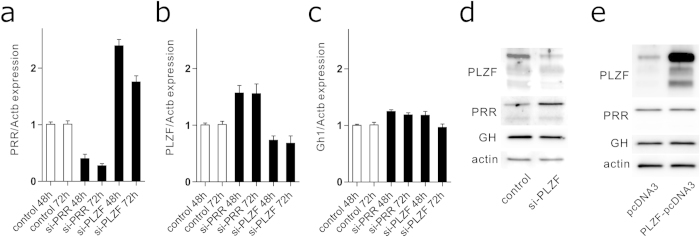
No significant association of the PLZF signal transduction pathway in GH expression. **a–c** GH3 cells were transfected with scramble control si-RNA (control), PRR si-RNA (si-PRR), or PLZF si-RNA (si-PLZF) for 48 h. PRR, PLZF, Gh1 and Actb mRNA levels were quantified using real-time RT-PCR. **d** GH3 cells were transfected with either scramble control siRNA (control) or PLZF si-RNA (si-PLZF) for 48 h, and cell lysates were subjected to immunoblotting using anti-PLZF, anti-PRR, anti-GH, or anti-actin antibodies. **e** GH3 cells were transiently transfected with either control empty vector (pcDNA3) or PLZF-expressing vector (PLZF-pcDNA3) for 48 h, and cell lysates were subjected to immunoblotting using anti-PLZF, anti-PRR, anti-GH, or anti-actin antibodies. Representative blots are shown. Full-length blots were shown in supplementary figure 3.

Because transfection with PRR siRNA increased the intracellular protein levels of both PLZF and GH by 48 h, we tested whether PLZF mediates GH expression. PRR suppression with PRR siRNA increased PLZF mRNA levels (Fig. 4a,b), and transfection of PLZF siRNA induced partial suppression of PLZF mRNA and significant upregulation of PRR mRNAs (Fig. 4a,b). Gh1 expression was unaltered by suppressing either PRR or PLZF (Fig. 4c). Consistent with these gene expression results, western blot analysis revealed that PLZF suppression upregulated PRR protein without affecting GH levels (Fig. 4d). Furthermore, transient transfection of GH3 cells with PLZF-pcDNA3 neither affected PRR nor GH protein levels (Fig. 4e). These data negate the possibility that accumulation of intracellular GH protein after suppressing PRR is mediated via the PLZF transduction pathway.

### Role of PRR for stability of V-ATPase

**Figure 5:**
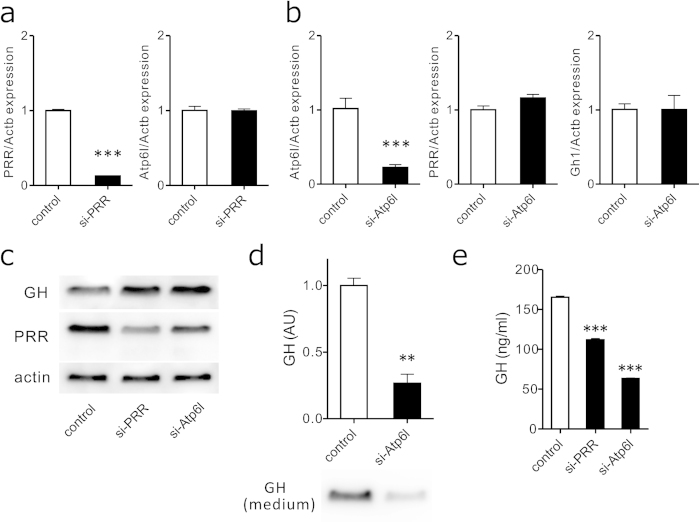
Reduction in GH release by Vo subunit instability. **a** GH3 cells were transfected with either scr**a**mble control siRNA (control) or PRR si-RNA (si-PRR) for 48 h, and PRR, Atp6l and Actb mRNA levels were quantified using real-time RT-PCR. **b** GH3 cells were transfected with either scramble control siRNA (control) or Atp6l si-RNA (si-Atp6l) for 48 h, and Atp6l, PRR, Gh1 and Actb mRNA levels were quantified using real-time RT-PCR. **c** GH3 cells were transfected with either scramble control siRNA (control), PRR si-RNA (si-PRR), or Atp6l si-RNA (si-Atp6l) for 48 h, and cell lysates were subjected to immunoblotting using anti-GH, anti-PRR, and anti-actin antibodies. **d** GH3 cells were transfected with either scramble control siRNA (control) or Atp6l si-RNA (si-Atp6l) for 48 h, and cell lysates were subjected to immunoblotting using an anti-GH antibody. A representative immunoblot (lower panel) and quantification of GH protein levels normalized to WST-1 absorbance (n = 3, upper panel) are shown. Each column and bar represents means ± SEM. **P < 0.01. **e** Culture supernatant from GH3 cells transfected with either scramble control siRNA (control), PRR si-RNA (si-PRR), or Atp6l si-RNA (si-Atp6l) for 60 h were subjected to GH ELISA, and GH levels were measured (n = 4). Each column and bar represents mean ± SEM. ***P < 0.001. Full-length blots were shown in supplementary figure 4.

We next assessed the PRR requirement for V-ATPase stability in GH3 cells by evaluating the relationship between PRR and Atp6l, a subunit of the V-ATPase Vo domain which has a putative bafilomycin A1- binding pocket^35^ and is selectively suppressed after ablation of the PRR gene (Atp6ap2)^11^. PRR knockdown did not affect Atp6l mRNA levels (Fig. 5a), and Atp6l suppression did not influence expression of PRR or Gh1 either (Fig. 5b). However, western blot analysis revealed that suppression of PRR or Atp6l resulted in upregulated intracellular GH protein levels (Fig. 5c), as well as significantly (*P* < 0.01) decreased GH protein levels detected in cultured media (Fig. 5d). GH released into GH3 cell culture supernatant was accurately measured using specific ELISA after treating cells with scrambled control siRNA, PRR siRNA, and Atp6l siRNA. Both suppression of PRR and Atp6l significantly (*P* < 0.001) reduced GH secretion from GH3 cells (Fig. 5e). Taken together, these results indicate that instability of V-ATPase impaired GH release without affecting GH expression.

## Discussion

The initial remarkable finding from the current study was an unexpected close association of PRR expression in GHomas with GH oversecretion. Surgically resected GHomas highly expressed PRR compared with the remaining human pituitary adenomas. The majority expressed a significant amount of prorenin as well. However, circulating GH/IGF-1 levels in our acromegalic patients were remarkably increased as PRR expression in GHomas intensified, although the levels were not associated with immunoreactive prorenin, suggesting a pivotal role for PRR in GH secretion, but not for prorenin. These data prompted us to investigate whether PRR regulates GH secretion independently from its classical receptor stimulation pathway induced by prorenin.

The clinical data have been strongly supported by our subsequent *in vitro* study using rat GHoma-derived cells, GH3, in which PRR clearly regulated GH release. Suppressed PRR expression by transfecting PRR siRNA into GH3 markedly decreased GH secretion in culture medium and accumulated GH protein levels within the cells. Proton transport into intracellular organelles is primarily mediated by V-ATPase, and the passage of secretory proteins through the Golgi complex involves translocation through an acidic compartment. PRR acts as an adaptor between Wnt receptors and V-ATPase complex to mediate Wnt signaling^17^. In the present study, treatment of GH3 with bafilomycin A1, which disrupts intracompartmental proton gradients, also reduced GH release and elevated intracellular GH protein levels. Further, knockdown of Atp6l, a component of the Vo domain V-ATPase, dramatically decreased GH secretion. Atp6l is a bafilomycin A1 binding subunit^35^, an enzyme complex that critically regulates vesicular acidification, and is down-regulated after deletion of the PRR gene^11^. Inhibition of PRR or Atp6l, as well as treatment with bafilomycin A1, is expected to cause impaired cytosolic acidification in GH3 cells. Because the passage of secretory proteins through the Golgi complex involves translocation through an acidic compartment, it is expected that both inhibition of PRR/Atp6l and bafilomycin A1 treatment results in defective vacuolar proton pumps, which further leads to an impaired constitutive- and regulated-secretory pathway. These data indicate a mediatory role for PRR in GH release from pituitary tumor cells.

PRR was shown to mainly localize in subcellular organelles, such as the endoplasmic reticulum and Golgi, with only a small amount on the plasma membrane^8^,^18^. The present study confirmed PRR distribution in the Golgi apparatus and lysosomes in human pituitary tumors. In GH3 cells, PRR was co-expressed with the Golgi marker GM130 and the ER marker PDI, and only partly with the plasma membrane marker Na^+^/K^+^-ATPase. Results showing intracellular localization of PRR argue against the involvement of prorenin, the ligand of full-length PRR that is expressed on the surface of plasma membrane.

Acidification-independent roles of V-ATPase in secretion and membrane fusion have been proposed^36^,^37^,^38^. In addition to accessory subunits of the Vo domain, Ac45 and a3 have been implicated in the regulatory function of secreting POMC and insulin, respectively^39^,^40^. Another subunit, a1 (V100) regulates the formation of SNARE complexes, and its disruption results in impaired neurotransmitter release^41^. The a3 isoform is expressed in membranes of insulin-containing secretory granules of pancreatic islets, while circulating insulin levels are reduced in mice carrying the a3 null mutation^40^. In the study, insulin secretion was not affected by 10 nM bafilomycin A1 treatment in βHC9 cells derived from hyperplastic mouse islets. A higher concentration of 500 nM bafilomycin A1 has been shown to inhibit both constitutive and regulated secretory pathways by blocking the vacuolar proton pump in transfected GH3 cells^42^. Marked suppression of GH release from GH3 cells in the present study was evident at 100 nM bafilomycin A1, but not with 10 nM (data not shown). Such contradictory results in the literature could be accounted for by the bafilomycin A1 concentration and/or the different cell types employed in each study.

In addition to V-ATPase, PRR has another direct protein-protein interaction partner termed PLZF, which binds to the PRR promoter to act as a repressor^18^. We also tested the possible contribution of this transcription factor in modulating PRR-mediated GH secretion. PLZF suppression caused PRR upregulation, while PRR suppression increased PLZF expression. However, neither knockdown nor over-expression of PLZF in GH3 cells affected GH production or release, indicating a negative role for PLZF in GH secretion. Thus, although PRR-mediated GH secretion is independent of PLZF, PRR acts as a repressor for PLZF expression. These results suggest the presence of a very short, negative feedback loop between PRR and PLZF.

In conclusion, our study revealed the preferential expression of PRR in human pituitary adenomas, in particular in GHomas, as well as in the rat GH3 cell line. The close association of high PRR expression in GHomas with markedly elevated circulating GH/IGF-1 levels in acromegalic patients may be explained by the regulatory mechanisms of GH secretion by PRR. Reduced GH secretion from GH3 cells, following transfection with PRR siRNA or treatment with a V-ATPase inhibitor, both of which impaired intracellular acidification, strongly suggests a functional role for PRR in GH secretion via V-ATPase.

## Methods

### Patients

Eighty-three patients with pituitary tumors (29 were male and 54 were female, mean age 48.2 ± 1.5 years), who underwent transsphenoidal surgery, were included in the present study. Thirty-three patients with acromegaly were diagnosed on the basis of typical clinical features, elevated basal serum GH levels (28.7 ± 8.1 ng/mL) obtained from mean of at least two preoperative values after overnight fasting, absent suppression of GH levels to <1 ng/mL after a 75-g oral glucose tolerance test (OGTT), higher IGF-I levels compared with those in age- and sex-matched control subjects, and the presence of pituitary tumor on brain magnetic resonance imaging. The pathological diagnosis of GH cell adenomas (GHoma) was established by positive immunostaining for GH.

This study was approved by the Ethics Committee of Toranomon Hospital, and all subjects provided written informed consent. All the methods, including immunohistochemical studies of pituitary tumor tissues obtained from surgery, were carried out in accordance with the approved guidelines and regulations, and performed after approval by the Committee.

### Cell culture and transfections

Rat pituitary tumor cells (GH3, purchased from the Japanese Collection of Research Bioresources Cell Bank, Osaka, Japan) were cultured in 82.5% Ham’s F10 medium with 15% horse serum and 2.5% fetal calf serum. Rat PLZF cDNA was inserted into the mammalian expression plasmid (pcDNA3.1/V5-His) and transfected with GH3 cells using Lipofectamine 2000 (Invitrogen, Carlsbad, CA, USA) according to the manufacturer’s instructions. The plasmid sequence was confirmed. The small interfering RNA (siRNA) duplexes against rat PRR, PLZF, and Atp6l were obtained from Ambion (Invitrogen). GH3 cells cultured in a six-well plate for 48–72 h were transfected with 10 nM of either a negative control, rat PRR, PLZF, or Atp6l siRNA duplexes using Lipofectamine RNAiMAX (Invitrogen).

### Real-time RT-PCR

Total mRNA was isolated from GH3 cells using TRIzol reagents (Invitrogen), reverse transcribed with the first-strand cDNA synthesis kit (Takara Bio Inc., Shiga, Japan), and quantified by a Chromo4 (Bio-Rad Laboratories, Hercules, CA, USA)-based RT-PCR protocol using KAPA SYBR (Nippon Genetics, Tokyo, Japan) as described^43^. Data were expressed as Ct values and used to determine ΔCt values, where ΔCt represents Ct of the target gene minus Ct of the housekeeping actin gene. The following primers were used for RT-PCR: PRR, forward primer 5′-tttcgtgtggctcatctcc-3′ and reverse primer 5′-gctaaattcattcgctaaagcac-3′, PLZF, forward primer 5′-ggaggagcagtgcctgaa-3′ and reverse primer 5′-ggtggcttctgtgtcattgtc-3′, Gh1, forward primer 5′-gatcactgagtggcgatgg-3′ and reverse primer 5′-gaaagcaccagcctcttga-3′, Atp6l, forward primer 5′-gtccgccatggtcttcag-3′ and reverse primer 5′-ggacttcatgatcagctctgg-3′, Actb, forward primer 5′-cccgcgagtacaaccttct-3′ and reverse primer 5′-cgtcatccatggcgaact-3′ (Eurofins MWG Operon, Tokyo, Japan). A comparative threshold cycle method was used.

### Immunoblotting

Cells were washed three times with phosphate-buffered saline (PBS), scraped from the culture dishes, and solubilized in RIPA buffer (Thermo Scientific, Lafayette, CO, USA) with 10 μM protein inhibitors (Thermo). The samples were then centrifuged at 20,000 g for 15 min, and the supernatants were collected. Protein determination was performed using the BCA assay kit (Pierce, Rockford, IL, USA). Samples were loaded on 4–20% gradient polyacrylamide gel (Bio-Rad) and transferred to PVDF membranes. After blocking in 10 mM Tris-HCl pH 7.4 and 150 mM NaCl (TBS) containing 5% non-fat milk, membranes were incubated with primary antibodies overnight at 4 °C. Primary antibodies for GH (1:500, R&D), PLZF (1:200, Santa Cruz Biotechnology, Santa Cruz, CA, USA), Atp6l (1:500, Abcam, Cambridge, MA, USA) and β-actin (1:1000, Santa Cruz Biotechnology) were used for the study. Polyclonal rabbit anti-human PRR (1:1000, ProteinExpress, Chiba, Japan), which recognizes extracellular domains, has been previously described^8^. After extensive washing, the membrane was incubated with peroxidase secondary antibody for 1 h at room temperature, and the complexes were detected using an enhanced chemiluminescence (ECLplus) system (GE Healthcare, Tokyo, Japan). The signals of each blot were visualized and quantitatively analyzed by ImageQuant LAS 4000 (GE Healthcare).

### Immunohistochemistry

Immunostaining was performed by the avidin-biotin-peroxidase complex methods using a Benchmark HX automated immunostainer (Ventana Medical Systems Inc., Tucson, AZ, USA) according to manufacturer’s instructions. An anti-PRR antibody (ProteinExpress) was used at a dilution of 1:50. An anti-prorenin polyclonal antibody was a kind gift from S. Hirose, which had been raised in rabbits as described^44^ using N-terminal 13 amino acid residue of human prorenin, and used at a dilution of 1:100. Hematoxylin was used as a counterstain. The resulting sections were blindly examined, without any prior knowledge of pituitary subtype, and were independently diagnosed by two expert pathologists. The immunostaining intensity in the cytoplasm was evaluated on a scale of 0–2 + .

### Immunofluorescence staining

GH3 cells were fixed in PBS containing 4% paraformaldehyde for 15 min, followed by permeabilization with PBS containing 0.2% Triton X-100 for 15 min. The cells were then blocked with 1% BSA in PBS for 30 min, incubated with primary antibody for 30 min, washed, and then incubated with secondary antibody for 30 min. Primary antibodies against PDI (1:100, Abcam), GM130 (1:100, Abcam), Na^+^/K^+^-ATPase α1 (1:50, Santa Cruz Biotechnology), and PRR (1:100, ProteinExpress) were used. Alexa 488-conjugated anti-rabbit antibody (1:2000, Molecular Probes, Eugene, OR, USA) and Alexa 546-conjugated anti-mouse antibody (1:2000, Molecular Probes) were used as secondary antibodies. For the labeling of acidic organelles, GH3 cells were incubated with LysoTracker Red (Invitrogen-Molecular Probes) for 30 min, and then fixed with 4% paraformaldehyde in PBS (pH 7.4). Signals were observed using an LSM510 confocal microscope system (Carl Zeiss, Jena, Germany).

### Cell proliferation assay

Cell number was assessed using the WST-1 Cell Proliferation Assay Kit (Takara), which marks metabolically viable cells, and the cells were read at 450 nm with a reference reading at 595 nm in an iMark Microplate Reader (Bio-Rad).

### Hormone assay

Cells were plated in quadruplicate wells, transfected with siRNA for 60 h, and the medium was changed to serum-free medium for 3 h. The conditioned medium was collected and centrifuged to remove debris, and subsequently frozen for ELISA measurements. Rat GH was measured using the GH ELISA kit (Invitrogen). Secreted hormone levels were normalized to WST-1 absorbance.

### Statistical analysis

Differences between groups were examined for statistical significance using the unpaired *t* test or Kruskal–Wallis test with the Dunn’s post hoc test, if appropriate. Data are presented as mean ± SEM, with *P* < 0.05 considered as statistically significant. All statistical analyses were performed using Prism 5.0 (GraphPad Software, La Jolla, CA, USA).

## Additional Information

**How to cite this article**: Tani, Y. *et al.* Regulation of growth hormone secretion by (pro)renin receptor. *Sci. Rep.* **5**, 10878; doi: 10.1038/srep10878 (2015).

## Supplementary Material

Supplementary Information
