# Supplementary material for: Regulation of growth hormone secretion by (pro)renin receptor
Source: Sci Rep. 2015 Jun 3;5:10878. doi: 10.1038/srep10878 (PMC4454151; doi:10.1038/srep10878)
Supplement: Supplementary Information [file srep10878-s1.pdf]

Supporting information

Regulation of growth hormone secretion by (pro)renin receptor

Yuji Tani[1], Shozo Yamada[2], Naoko Inoshita[3], Yukio Hirata[4], & Masayoshi Shichiri[1]

[1]Department of Endocrinology, Diabetes and Metabolism, Kitasato University School of Medicine, Kanagawa, Japan

[2]Hypothalamic and Pituitary Surgery Center, [3]Department of Pathology, Toranomon Hospital, Tokyo, Japan
[4]Institute of Biomedical Research and Innovation Hospital, Hyogo, Japan

Correspondence and requests for materials should be addressed to M.S. (shichiri@kitasato-u.ac.jp).

Key words: (pro)renin receptor, ATP6AP2, growth hormone, V-ATPase

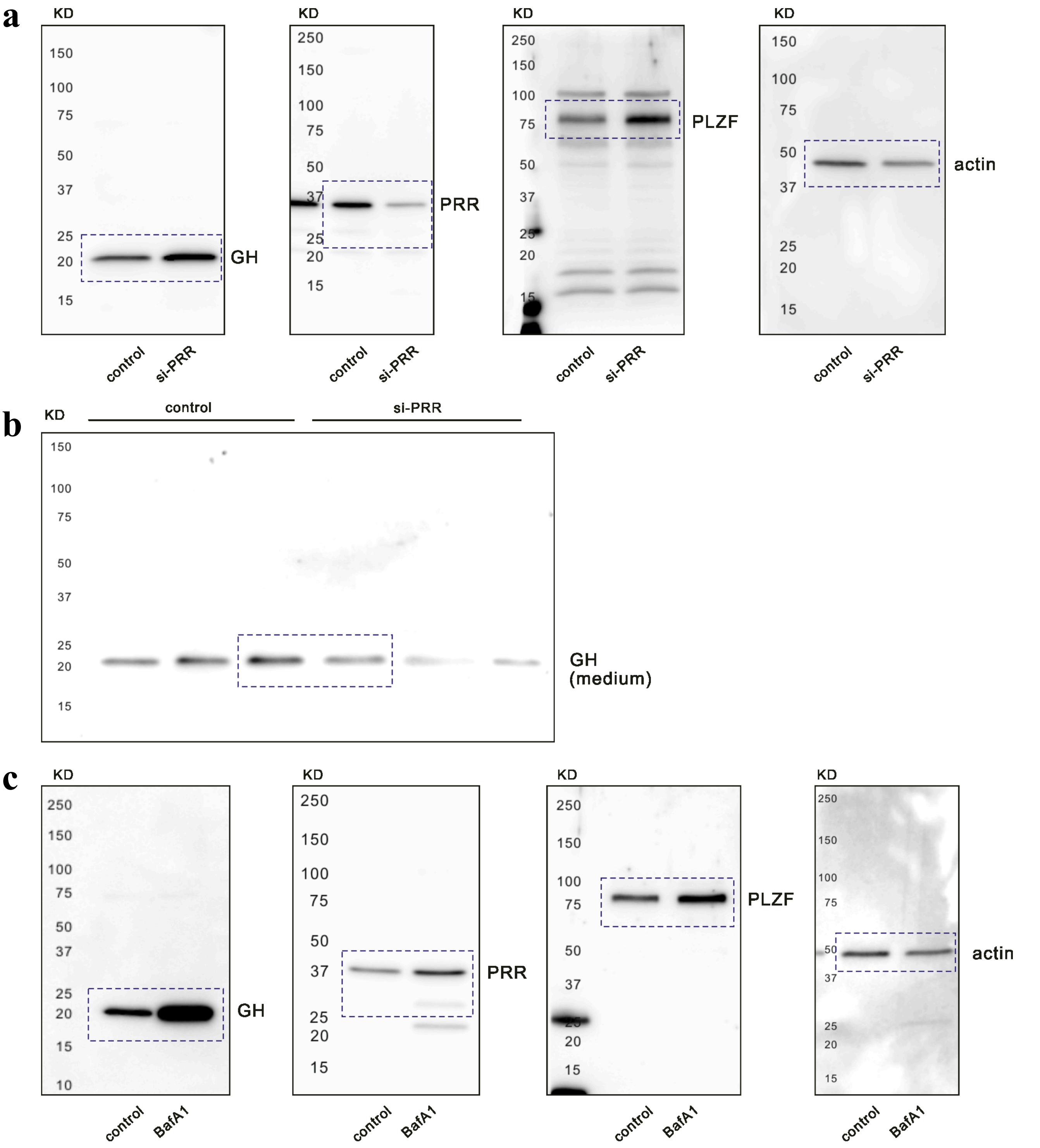

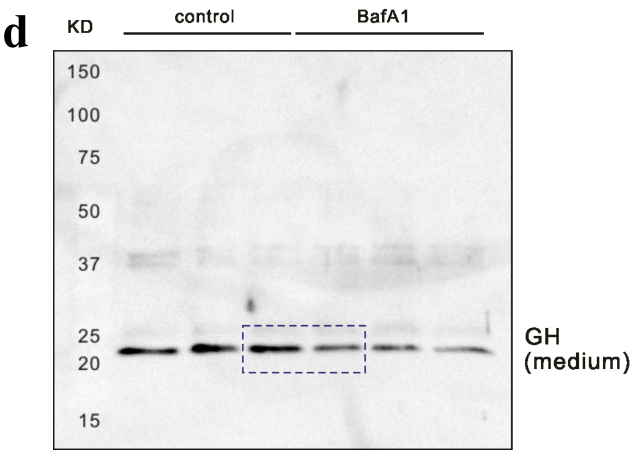

Supplementary Figure 1 **Full length blots for Figure 3a-d**
a, GH3 cells were transfected with either scramble control siRNA (control) or PRR si-RNA (si-PRR) for 48 h, and cell lysates were subjected to immunoblotting using anti-GH, anti-PRR, anti-PLZF, and anti-actin antibodies. **b**, Conditioned medium from cultured GH3 cells transfected with either scramble control siRNA (control) or PRR si-RNA (si-PRR) for 48 h was extracted and immunoblotted with an anti-GH antibody. **c**, GH3 cells were treated without (control) or with bafilomycin A1 (BafA1, 100 nM) for 24 h, and cell lysates were subjected to immunoblotting using anti-GH, anti-PRR, anti-PLZF, and anti-actin antibodies. **d**, Conditioned medium from cultured GH3 cells treated without (control) or with bafilomycin A1 (BafA1, 100 nM) for 24 h, and cell lysates were subjected to immunoblotting using an anti-GH antibody.

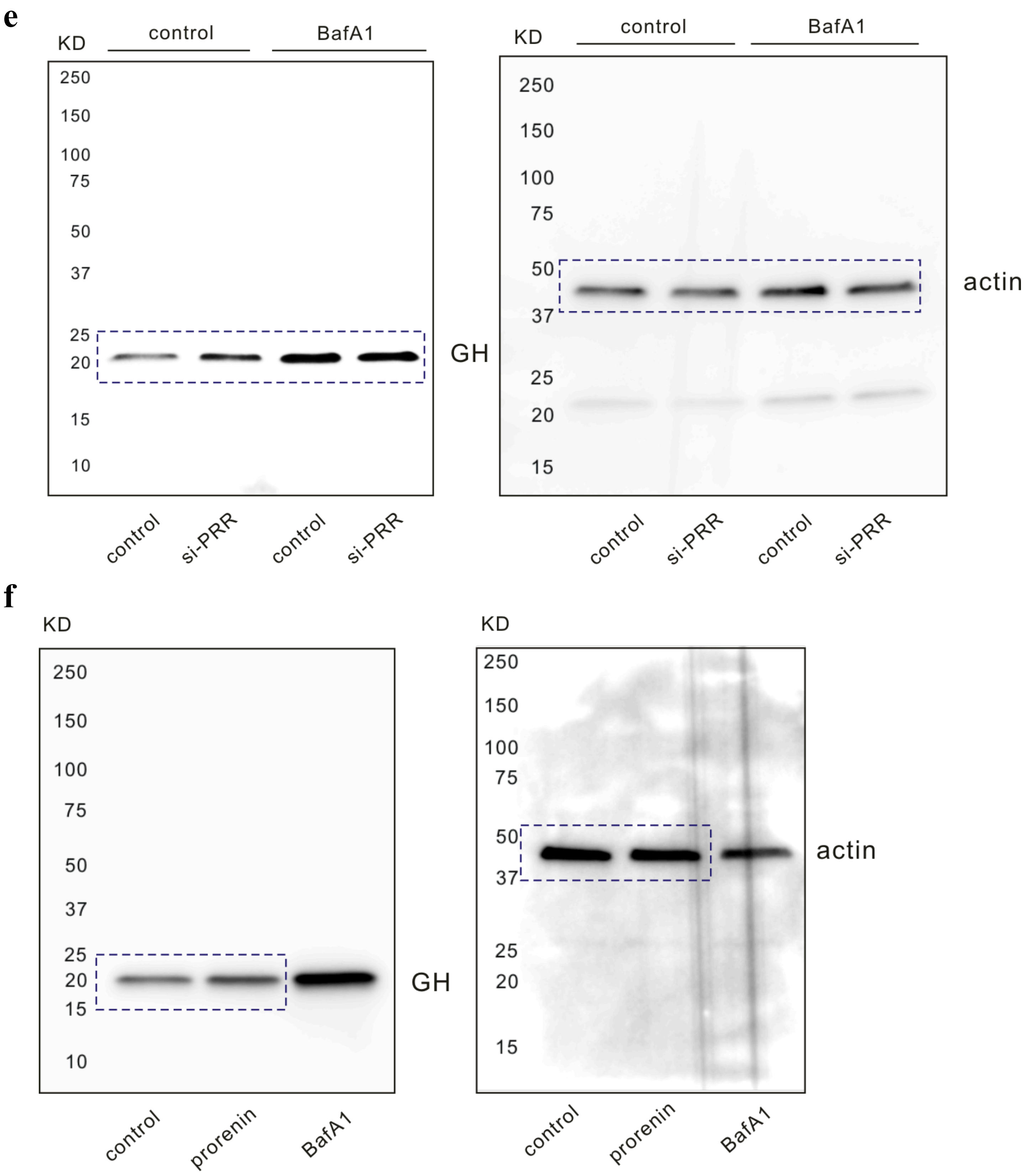

Supplementary Figure 2 Full length blots for Figure 3e, 3f

e, GH3 cells were transfected with either scramble control siRNA (control) or PRR si-RNA (si-PRR) for 48 h, further treated without (control) or with bafilomycin A1 (BafA1, 100 nM) for 9 h, and cell lysates were subjected to immunoblotting using anti-GH and anti-actin antibodies. **f**, GH3 cells were treated without (control) or with prorenin (5 nM) or with bafilomycin A1 (BafA1, 100 nM) for 24h, and cell lysates were subjected to immunoblotting using anti-GH and anti-actin antibodies.

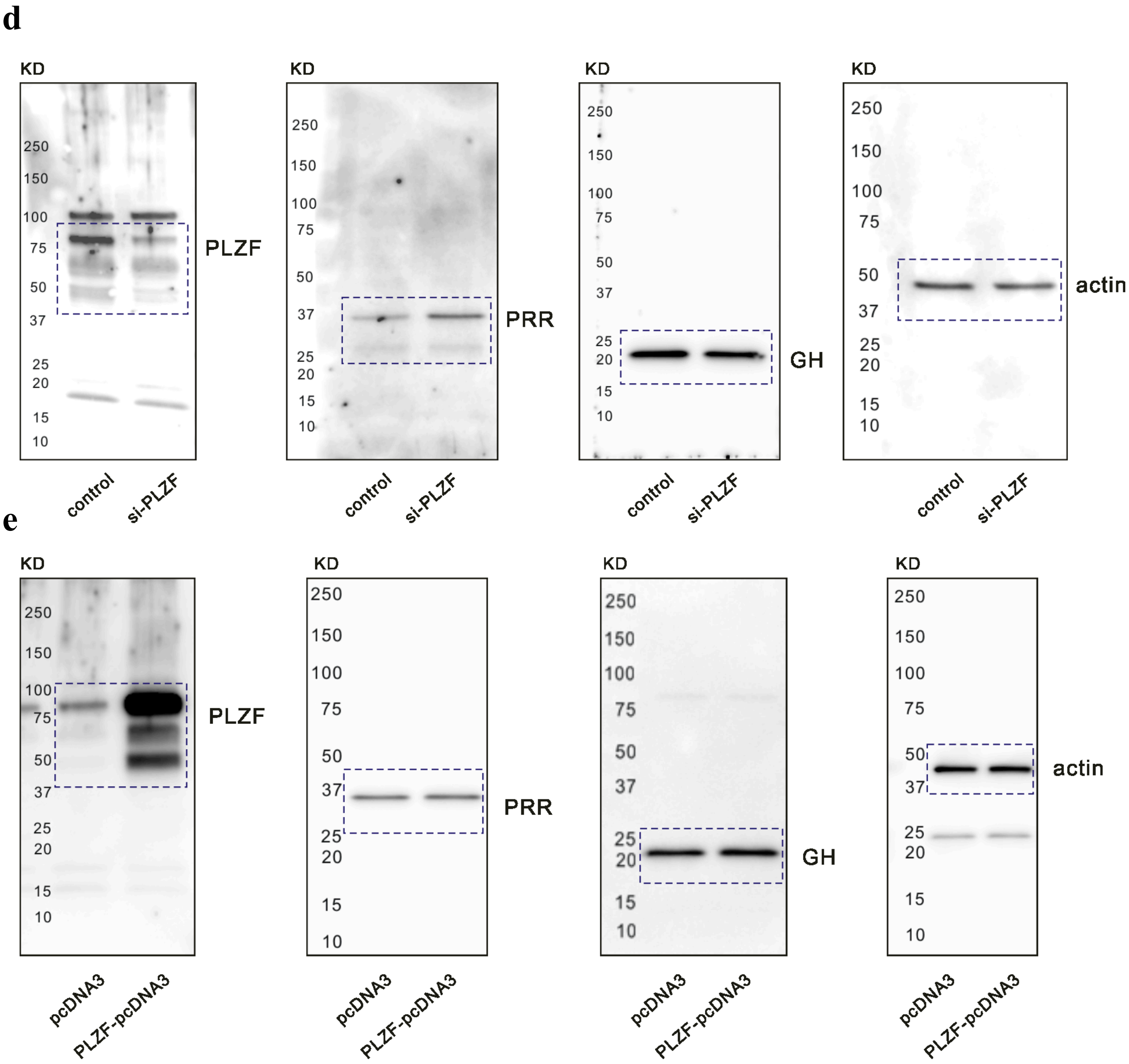

Supplementary Figure 3 **Full length blots for Figure 4**

d, GH3 cells were transfected with either scramble control siRNA (control) or PLZF si-RNA (si-PLZF) for 48 h, and cell lysates were subjected to immunoblotting using anti-PLZF, anti-PRR, anti-GH, or anti-actin antibodies. **e**, GH3 cells were transiently transfected with either control empty vector (pcDNA3) or PLZF-expressing vector (PLZF-pcDNA3) for 48 h, and cell lysates were subjected to immunoblotting using anti-PLZF, anti-PRR, anti-GH, or anti-actin antibodies.

c

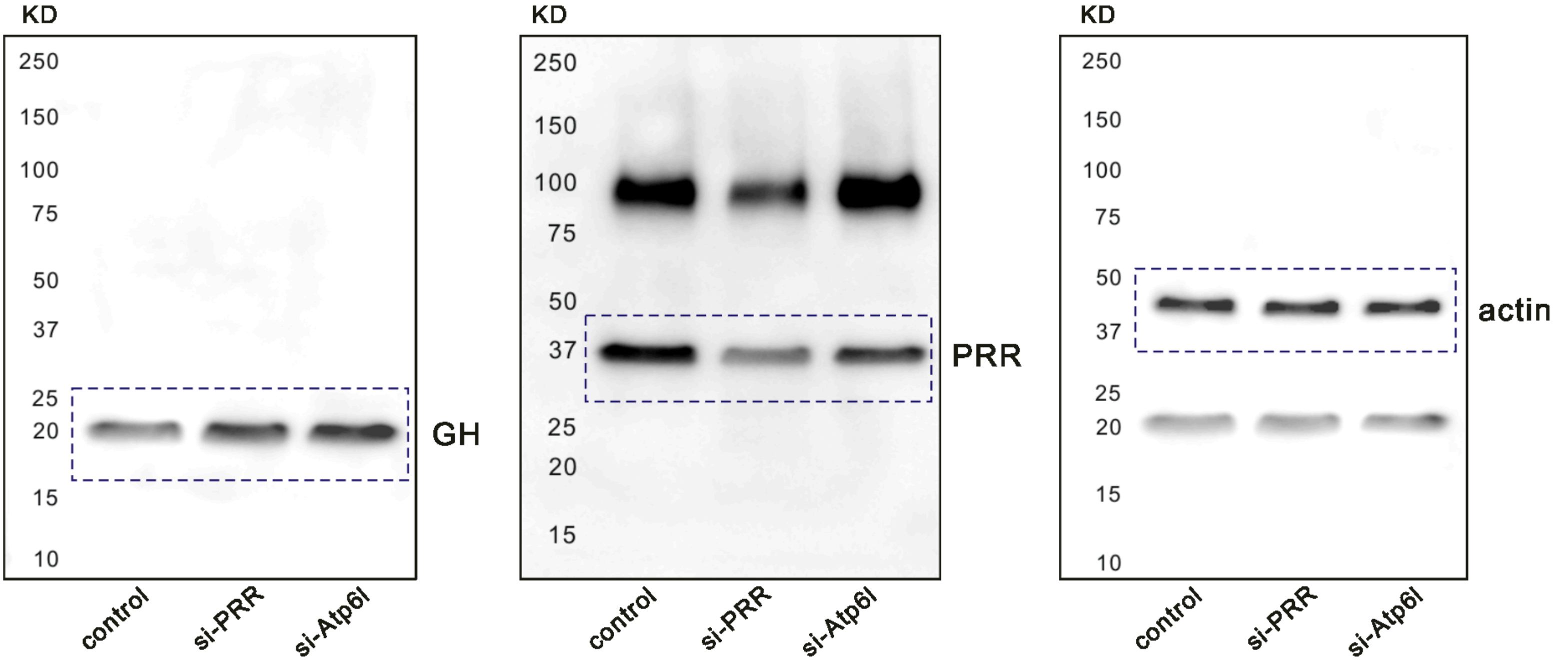

d

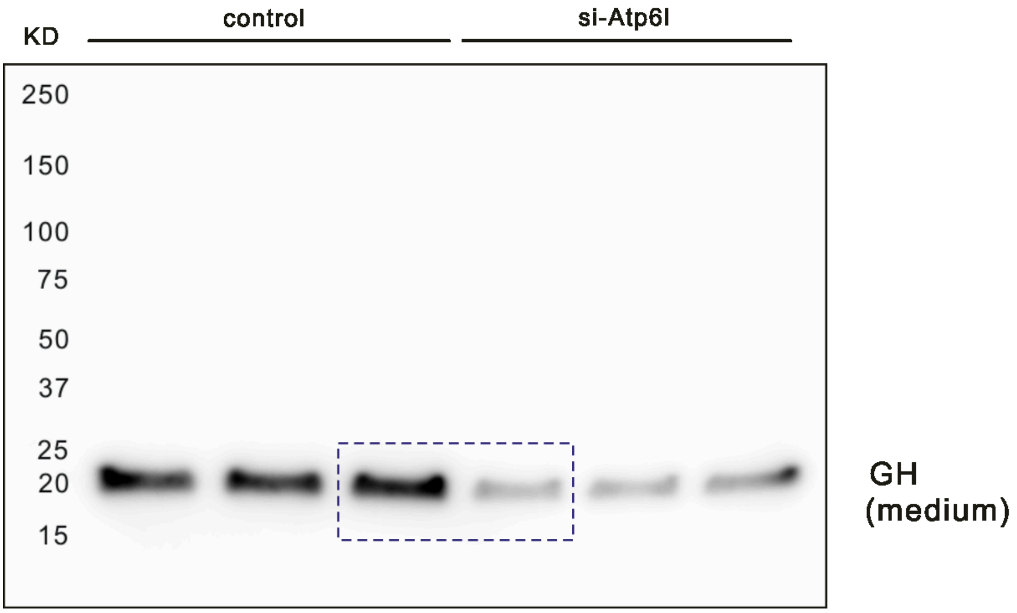

Supplementary Figure 4 **Full length blots for Figure 5**

c, GH3 cells were transfected with either scramble control siRNA (control), PRR si-RNA (si-PRR), or Atp6l si-RNA (si-Atp6l) for 48 h, and cell lysates were subjected to immunoblotting using anti-GH, anti-PRR, and anti-actin antibodies. **d,** GH3 cells were transfected with either scramble control siRNA (control) or Atp6l si-RNA (si-Atp6l) for 48 h, and cell lysates were subjected to immunoblotting using an anti-GH antibody.
